# Negative Gaseous Ions in Positive-Voltage Electrospray Ionization Mass Spectrometry

**DOI:** 10.1021/acs.analchem.5c08108

**Published:** 2026-03-03

**Authors:** Xing-Bo Wang, Ochir Ochirov, Bo-Cheng Ke, Noor Hidayat Abu Bakar, Chamarthi Maheswar Raju, Ioan Marginean, Pawel L. Urban

**Affiliations:** † Department of Chemistry, 34881 National Tsing Hua University, 101, Section 2, Kuang-Fu Rd., Hsinchu 300044, Taiwan; ‡ School of Criminal Justice, 205717 University of Baltimore, 10 W Preston St, LAP 515, Baltimore, Maryland 21201, United States

## Abstract

Here, we report a serendipitous observation of negative ions in positive-voltage electrospray ionization (ESI) mass spectrometry (MS). Several previous studies have demonstrated that electrospray operated with positive voltage can generate droplets carrying a negative net charge. Our spray current measurements on a counter electrode with concentric conductive rings indicate the presence of negative species within the charged aerosol produced by positive voltage electrospray. MS measurements performed in negative-ion mode captured negative ions generated by positive electrospray, both in the presence and absence of nebulizing gas. Analyte structure influences the ability of positive electrospray to generate the corresponding negative ions. The MS intensities produced by positive and negative ions were compared when positive and no voltage was applied to the electrospray. Possible mechanisms underlying these observations are discussed in the context of recent literature. These findings represent the first direct MS measurement of negative desolvated gaseous ions from standard positive electrosprays and provide new mechanistic insights that may inform future ESI development.

## Graphical abstract

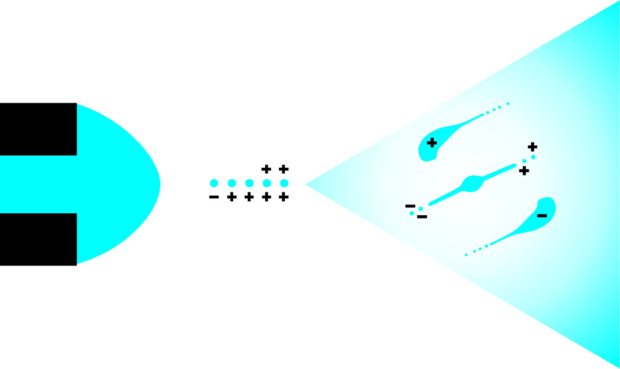

## Introduction

Introduced in the 1980s,^1–3^ electrospray ionization (ESI) mass spectrometry (MS) has enabled a wide range of applications.^4^ In a typical ESI-MS analysis, a liquid sample is infused through a conductive capillary emitter supplied with high voltage to generate charged droplets.^5^ The original droplets constantly shrink due to solvent evaporation and fission.^6,7^ Gas-phase ions eventually emerge from nanodroplets through a mechanism that is dependent on the analyte structure.^8^ Positive voltage is typically applied when positive ions are monitored by the mass spectrometer operated in the positive-ion mode. The implicit assumption is that positive ions are produced from positively charged droplets. Similarly, an electrospray operated with negative voltage is expected to produce negative droplets and the negative ions are monitored by the mass spectrometer in the negative-ion mode.

Image charge detection demonstrated that both droplets with positive and negative net charges can be produced when the electrospray is operated with positive high voltage.^9,10^ While the charge detection technique is useful for measuring charges of large particles such as microdroplets, MS detection of desolvated negative ions in positive ESI has not been demonstrated to date. High-speed imaging of the pulsating electrospray dynamics captured droplets moving toward the emitter, indicating that the charge they carried was of opposite polarity to the voltage applied to the electrospray.^11,12^ Our recent observations also show returning droplets in paper spray (see Figure S1). A minority of returning droplets were also detected by phase Doppler anemometry,^7^ as represented by a peak corresponding to negative velocities (see Figure 9 in ref 7). The negative velocities were interpreted as the result of the fission process rather than due to droplets carrying negative charge. Evidence for such returning droplets has also been provided by molecular dynamics simulations.^13^ The percentage of negative droplets reported in positive electrosprays varies from 1%^9^ to as much as 20%.^14^

When subjected to high electric fields, neutral droplets can deform symmetrically to eject both positively and negatively charged jets. This phenomenon was used in the so-called field-induced droplet ionization (FIDI) to concurrently generate both positive and negative droplets.^15^ FIDI took advantage of both the positive and negative droplets for MS measurements. Indirect evidence also suggests that some droplets generated by electrospray can undergo similar symmetric deformations.^16^ Charged droplets levitated in an electrohydrodynamic balance also exhibited symmetric deformation as solvent evaporation brought them close to the Rayleigh charge limit.^17^ These symmetric fission mechanisms may occur within the electrospray plume, with net neutral droplets generating both positive and negative progeny droplets, and positive droplets producing mostly positive progeny droplets.

The fate of the negative droplets generated by positive electrospray is unclear. Besides detection, their distribution within the electrospray plume has not been studied. It also remains unknown whether they give rise to negative ions that can be captured by MS. Here we radially profile the electrospray plume to provide evidence that negative species reach a counter electrode. Additionally, we use the mass spectrometer in the negative-ion mode to demonstrate that we can capture negative ions while applying a constant positive voltage to the electrospray emitter.

## Experimental Section

### Chemicals

Methanol (LC–MS grade) was purchased from Merck (Darmstadt, Germany). Water (LC–MS grade) was purchased from Fisher Scientific (Waltham, MA, USA). Ammonium hydroxide (30–33%, w/w) was purchased from Honeywell (Charlotte, NC, USA). Formic acid (≥98%) was purchased from Thermo Fisher Scientific (Fair Lawn, NJ, USA). l-glutathione (abbreviated as GSH; reduced ≥ 98%) and Gly-His (GH) were purchased from Sigma-Aldrich (St. Louis, MO, USA). Custom-synthesized peptides HPF, GGY, and TYS (H: histidine; P: proline; F: phenylalanine; G: glycine; T: threonine; Y: tyrosine; S: serine) were purchased from BioAb (New Taipei City, Taiwan). l­(+)-Glutamic acid (Glu; 99%) was purchased from Acros Organics (Geel, Belgium).

### Spray Current Measurements

A peristaltic pump (PF102; Yotec Instruments, New Taipei City, Taiwan) delivered aqueous solution containing 25% (v/v) methanol and 0.5% (v/v) formic acid with a flow rate of 8 μL min^–1^ to an ESI capillary (tip I.D.: 100 μm; tip O.D.: 270 μm; length: 82.5 mm; part no.: 225-14915; Shimadzu, Kyoto, Japan). A high-voltage power supply (MPS10P10/24/VCC; Spellman, Hauppauge, NY, USA) was used to apply constant +3 kV directly to the capillary without any modulation. The capillary was positioned 10 mm from the counter electrode described below, pointing directly at its center. These conditions ensure electrospray operation in the pulsating regime.^18^

Spray current was measured with a custom-built collector, hereafter referred to as a “structured Faraday plate” (Figure 1A). The collector comprised a central copper disk and ten copper concentric rings photolithographed onto a 70 × 70 × 1.6 mm Bakelite substrate (PS114.165-1.6; Kinsten Industrial Corp., New Taipei City, Taiwan). The central disk had a diameter of 6 mm and it was surrounded by ten 2 mm-wide conductive concentric rings, separated by 1 mm nonconductive gaps. The rings were numbered from 1 to 10, with 1 being the innermost ring. The central disk electrode and ten concentric ring electrodes were each connected to ∼8 mm long wires soldered to the electrodes through drilled holes (⌀ = 0.5 mm). The electrodes were connected to two coaxial cables fitted with BNC connectors. One cable was permanently attached to the central disk electrode, while the other cable was sequentially attached to the different ring electrodes. The measurements were performed with a two-channel digital oscilloscope (Analog Discovery 2; Digilent, Pullman, WA, USA), and the current was calculated as the ratio between the measured voltage and the input impedance of the oscilloscope (1 MΩ).

The noise recorded with no electrospray voltage applied (approximately −1.2 ± 0.2 nA) was subtracted from all spray current values to ensure a current baseline around 0 nA. The spray current frequency values were obtained using the fast Fourier transform (FFT) tool in OriginPro (version 9.8; OriginLab, Northampton, MA, USA). The spray current peak-to-peak amplitudes were estimated to represent the span between local maxima and minima across 12 consecutive cycles.

### Mass Spectrometric Measurements

Mass spectrometric measurements were performed using a triple quadrupole mass spectrometer (LCMS-8030; Shimadzu) coupled to a flow injection system. Samples were introduced via the sample loop of a 6-port valve integrated into a high-performance liquid chromatograph (Chromaster 5110; Hitachi, Tokyo, Japan) used as a flow injector (cf. ref 19). Each sample was carried to the ESI emitter as a discrete plug within the solvent stream delivered by the chromatograph. The ESI interface consisted of a stainless steel capillary emitter (100 μm tip i.d., 270 μm tip o.d.; cat. no. 225-14915; Shimadzu) and a tee (thru hole, 0.02 in part no. P-727; Idex Health & Science, Oak Harbor, WA, USA) enabling optional application of nebulizing gas (nitrogen) at a pressure of 40 psi. The emitter protrusion against the outer PTFE tubing (I.D. ∼0.5 mm; O.D. ∼1.5 mm; length 30 mm) was ∼2 mm. The emitter was placed parallel to the MS inlet axis and at a distance of ∼6 mm from the inlet. It was connected to a positive-polarity high-voltage power supply (MPS10P10/24/VCC; Spellman). The carrier solution was 25% (v/v) aqueous methanol solution with 0.178 M NH_3_(aq). It was pumped at a flow rate of 50 μL min^–1^ when using nebulizing gas or 35 μL min^–1^ without nebulizing gas. The sample injection volume was 10 μL. Measurements were conducted under two conditions: with the emitter biased at +3.8 kV, and with no voltage applied. The drying gas flow rate was 3 L min^–1^. The desolvation line (ion transfer capillary) was maintained at a temperature of 250 °C. The heated block temperature was 400 °C. The mass spectrometer was operated in the negative or positive-ion mode depending on the goal of each experiment. Q3 scan (*m*/*z* range 100–1200) and selected ion monitoring (SIM) were used.

## Results and Discussion

### Spray Current Measurements

Conductive counter electrodes have been used to detect the current generated by electrospray for more than a century.^20^ The counter electrodes are typically fabricated from solid metal and they collect the entire current generated by the electrospray. A linear array of detectors was previously used for spatial profiling of the electrospray current and simultaneous mass spectrometric measurements.^21^ Charge collectors composed of concentric conductive rings were also used to profile ion distribution in ion mobility spectrometry^22–24^ and to spatially profile the current generated by electrospraying ionic liquid ferrofluid in high vacuum.^25^ Here, we used the structured Faraday plate illustrated in Figure 1A to profile the positive electrospray plume.

**Figure 1:**
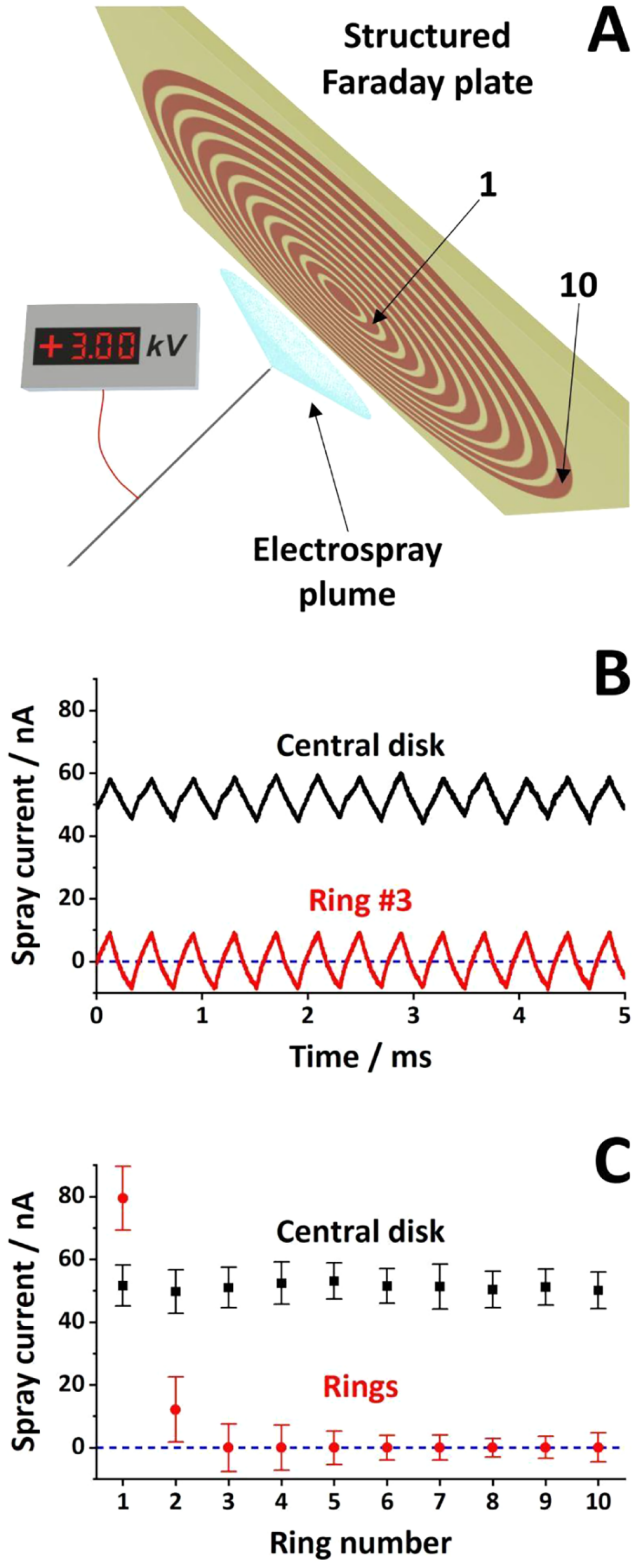
Spray current measurements with the structured Faraday plate detector: (A) setup layout; (B) simultaneous spray current measurements for the central disk (black trace) and the concentric ring #3 (red trace); (C) summary of spray current measurements on the structured Faraday plate with the whiskers representing the span between minima and maxima of the pulsating spray current. ESI emitter voltage: +3 kV. Sample: aqueous methanol (25%, v/v) with 0.5% (v/v) formic acid. Sample flow rate: 8 μL min^–1^.

The spray current on the central disk was continuously monitored on the first oscilloscope channel, while the second channel sequentially measured the current on each of the ring electrodes. The black trace in Figure 1B is representative for a central disk current measurement. The electrospray operated in the natural pulsating regime is characterized by repeated formation of a Taylor cone followed by its collapse to a rounded meniscus.^26^ The black symbols in Figure 1C reflect the measurement repeatability on the central disk. The whiskers in this figure indicate the peak-to-peak amplitude in the spray current. Based on ten measurements, the average spray current on the central disk was 51.2 ± 1.0 nA, the oscillation peak-to-peak amplitude was 12.5 ± 1.2 nA (indicated by the length of the whiskers), and its frequency was 2.9 ± 0.3 kHz.

The spray current oscillations are known to correlate with successive Taylor cone formation and its subsequent collapse.^18,27,28^ During the liquid ejection phase, the electrospray generates a charged aerosol that travels toward the counter electrode. Highly mobile species (ions and small, highly charged droplets) reach the counter electrode first. The spray current quickly increases and goes through a local maximum. Based on reanalysis of images, the liquid ejection phase was estimated to last ∼35–45% of the pulsation cycle.^12,27,28^ As the electrospray progresses into the other phases of the pulsating regime (relaxation, liquid accumulation, cone formation), the spray current is expected to drop. In most experiments, the current remains significantly above the baseline, suggesting that consecutive charged aerosol clouds spread out and overlap by the time they reach the counter electrode. The high mobility species—ejected during the liquid ejection phase—likely catch up with lower mobility species generated during the previous pulsation.

The red trace in Figure 1B shows the spray current measured on concentric ring #3, which is representative for oscillating currents collected on the ring electrodes. The current was in phase with that measured simultaneously on the central disk. The average current on ring #3 was indistinguishable from the baseline, with a peak-to-peak amplitude of 15.2 nA. The series of spray current measurements on the ten rings were summarized as red symbols in Figure 1C. The full set of paired spray current measurements—the central disk and the respective ring—is presented in Figure S2. The average current decreased radially from 79.4 nA (ring #1) and 12.1 nA (ring #2) to values close to the baseline on all remaining rings. The peak-to-peak amplitudes decreased less drastically from ∼21 nA (for rings #1–2) to ∼8 nA (for rings #6–10). Even though the average current on the central disk (51.2 nA) was lower than that on the second ring (79.4 nA), the current density (1.8 nA mm^–2^ for the central disk, 1.3 nA mm^–2^ for ring #1, 0.1 nA mm^–2^ for ring #2, and zero for rings #3–10) aligns with the current understanding of charge distribution within the electrospray plume.^21,29^ Additionally, electrospray plume expansion and the higher droplet concentration in its core, with a gradual decrease toward the outskirts, were visualized using scattered blue laser pointer light (Figure S3).

While positive currents are normally recorded for positive electrosprays using solid counter electrodes, it is particularly striking that the momentary current on outside rings #3–10 (the red trace in Figure 1B and the red symbols in Figure 1C) reached negative values. Such symmetrical oscillations around the baseline have never been reported, likely because solid counter electrodes capture the entire spray current, and they have no radial profiling capability. This oscillating spray current can be explained as (1) the conduction current resulting from positive and negative species alternately impinging on the electrodes, and (2) artifacts due to the current electromagnetically induced on the electrodes by the change in magnetic flux produced by the alternating current in the neighboring electrodes. To evaluate the induced current on ring #1, we applied a sinusoidal waveform to the central disk and measured the signal induced on ring #1. The sinusoidal signal (DC offset: 65 nA, peak-to-peak amplitude: 20 nA, frequency: 2 kHz) resembled the signal we typically measure on the central disk. While not entirely absent, the current induced in ring #1 (the black trace in Figure S4) had a peak-to-peak amplitude of 2.4 nA, which was almost 1 order of magnitude smaller than that of our typical current measurements (the red trace in Figure S4). We concluded that electrospray—not electromagnetic induction—contributed most of the current measured on the outside rings. We interpret these negative spray current values as an indication of negatively charged droplets and/or ions. Such negative species might form within the entire plume but were not registered as negative current on the central disk, rings #1 and #2 of the structured Faraday plate due to the prevalence of positive species in those regions. The experiments described here involved the electrospray operated in the pulsating regime. We have no experimental proof that negative droplets would also be formed in the cone-jet electrospray.

While the positive species are expected to move toward the counter electrode, the motion of negative species against the electric field is counterintuitive. A variety of forces act upon charged species traversing the space between the emitter and the counter electrode. These include an electric force (due to the applied voltage), aerodynamic drag forces (due to the interaction with neutral species), and electrostatic forces (due to space charge and image charges on the counter electrode).^30^ The electric field accelerates the positive species toward the counter electrode and the negative species in the opposite direction. The interaction with the neutral species (experienced as aerodynamic drag force) affects the motion of both positive and negative species. The positive species—present in the majority—are slowed down, and they provide the neutral species (gas molecules and solvent vapor) momentum toward the counter electrode. Vapor motion toward the counter electrode was observed experimentally within the electrospray plume.^31^ As charged species approach the counter electrode, they also experience an increasing attractive force due to the image charge. Although the movement of negative species against the electric field may be unexpected, their detection at the counter electrode can be explained by the aerodynamic drag force exerted by this flow of neutral species. To extend Kebarle’s metaphor describing the electrospray as an electrochemical cell of a special kind,^32^ the electrospray plume could be seen as moving toward the counter electrode due to an electroosmotic flow of a special kind.

### Ion Current Measurements

Negative ions can be detected in ESI-MS by applying constant negative voltage to the electrospray emitter, by rapidly switching emitter voltage polarity (alternating polarity mode),^33^ or by using a bipolar ESI source design.^34^ Although negative ions have not been detected with the electrospray operated with positive voltage, the generation of negative droplets by positive electrospray and the detection of negatively charged species on the counter electrode suggest that negative ions may form. Hence, we aimed to verify whether negative ions could be produced while applying a constant positive voltage to the electrospray emitter. Collecting data in the negative-ion mode while applying positive voltage on the electrospray is not possible using the standard ESI source of commercial mass spectrometers. Therefore, we fitted the ion source compartment with a house-built emitter assembly and supplied positive voltage from an external power supply while the mass analyzer was set to operate in the negative-ion mode. Surprisingly, deprotonated analytes (originated from positive electrospray) were detected in the negative-ion mode. These observations were made both with and without nebulizing gas (Figures 2 and 3). As expected, protonated analytes were recorded for the same samples in the positive-ion mode (Figures S5 and S6). Positive ion production was more effective than negative ion production by a factor of ∼30 (without nebulizing gas) or ∼300 (with nebulizing gas). Detection of negative ions for positive electrospray corroborates prior findings of negatively charged species within the plume.

**Figure 2:**
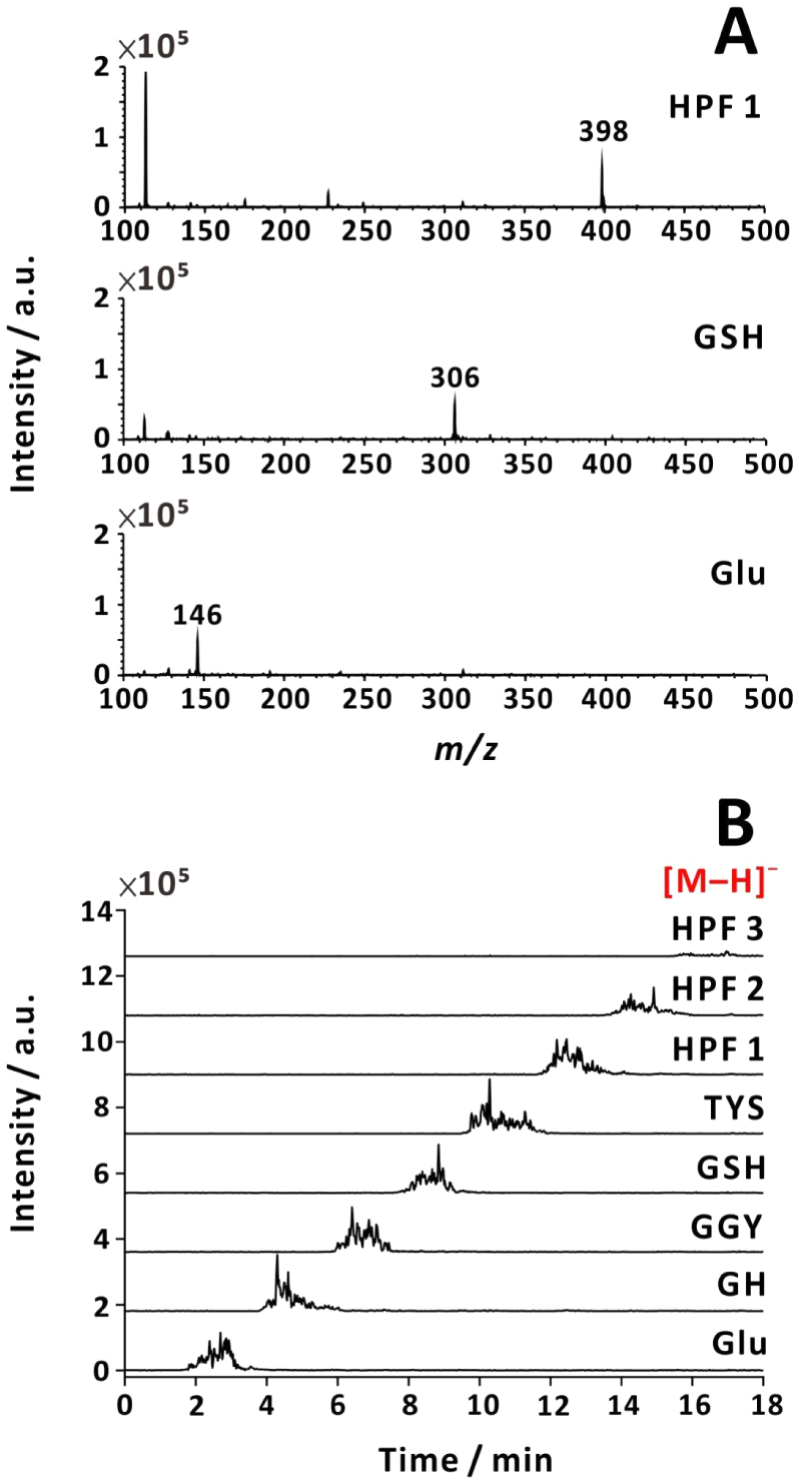
Negative-ion mass spectra (A) and currents recorded in SIM mode (B). Positive voltage (+3.8 kV) was applied to the emitter, and no nebulizing gas was used. Analytes: (1) HPF (*m*/*z* = 398); (2) GSH (*m*/*z* = 306); (3) l-glutamic acid (*m*/*z* = 146). Sample flow rate: 35 μL min^–1^. Sample solution: 80 μM analyte in 25% (v/v) aqueous methanol solution with 0.178 M NH_3_(aq).

**Figure 3:**
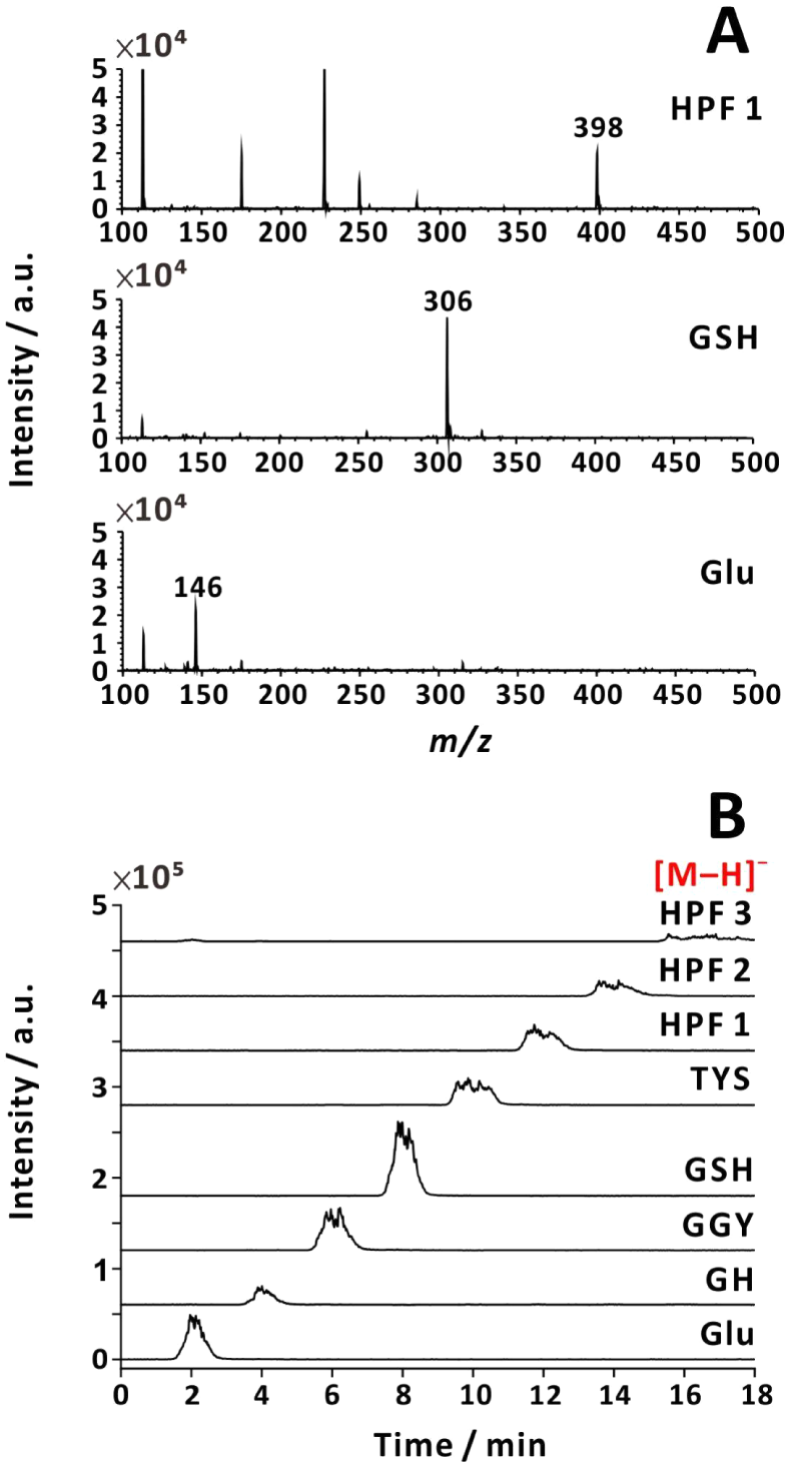
Negative-ion mass spectra (A) and currents recorded in SIM mode (B). Positive voltage (+3.8 kV) was applied to the emitter, and nebulizing gas was used (40 psi). Analytes: (1) HPF (*m/z =* 398); (2) GSH (*m*/*z* = 306); (3) l-glutamic acid (*m*/*z* = 146). Sample flow rate: 50 μL min^–1^. Sample solution: 80 μM analyte in 25% (v/v) aqueous methanol solution with 0.178 M NH_3_(aq).

In addition, both negative and positive ions were recorded even if no voltage was supplied to pneumatically controlled spray (Figures 4 and S7), which is in agreement with the reports on sonic spray ionization,^35,36^ easy ambient sonic-spray ionization,^37^ and Venturi easy ambient sonic spray ionization.^38^ Note that different diffusion coefficients can lead to different temporal MS signal profiles, which can be explained by Taylor–Aris dispersion.^39^ The negative ion temporal signal (recorded in SIM mode) was integrated, and the areas were displayed for different analytes when positive voltage or no voltage was applied to the electrospray emitter (Figure S8).

**Figure 4:**
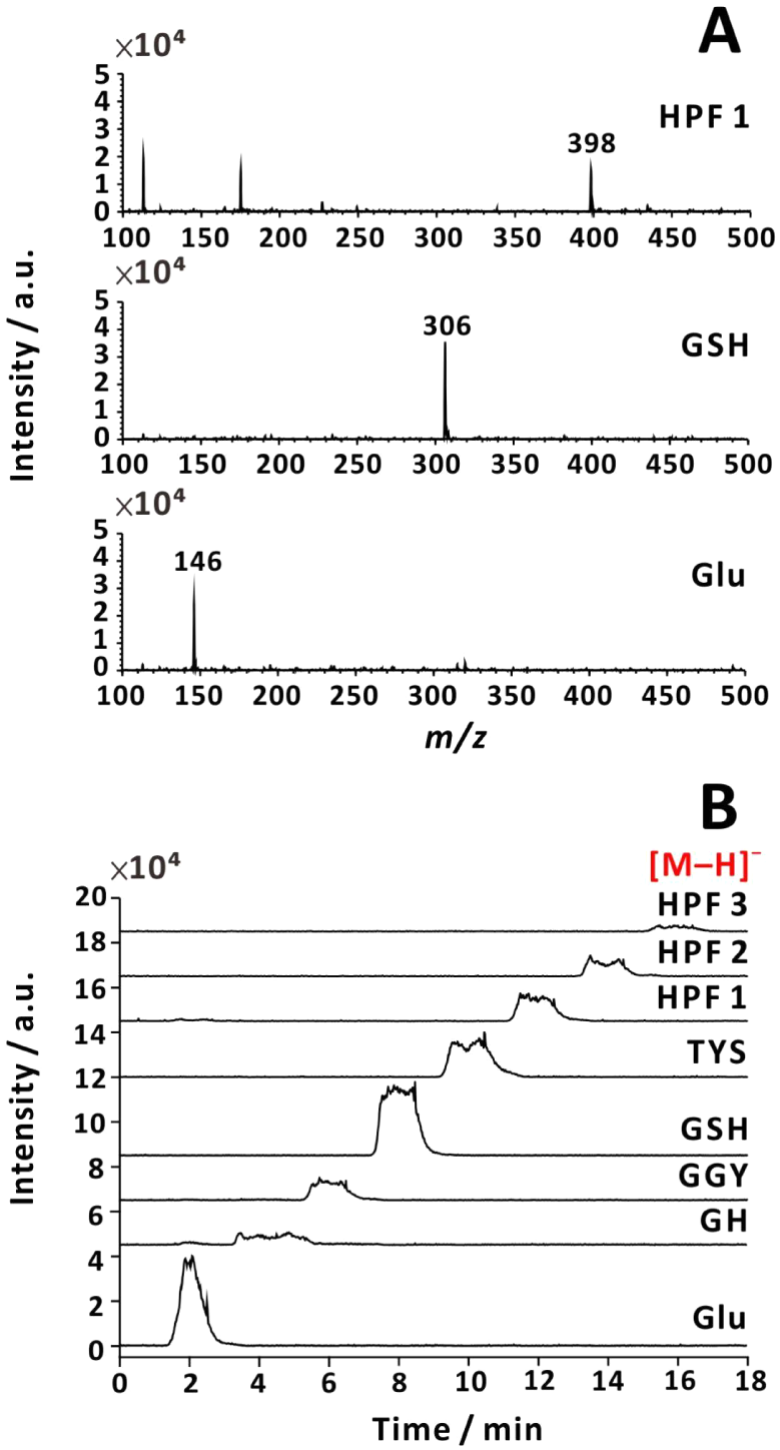
Negative-ion mass spectra (A) and currents recorded in SIM mode (B). No voltage (0 kV) was applied to the emitter, and nebulizing gas was used (40 psi). Analytes: (1) HPF (*m*/*z* = 398); (2) GSH (*m*/*z* = 306); (3) l-glutamic acid (*m*/*z* = 146). Sample flow rate: 50 μL min^–1^. Sample solution: 80 μM analyte in 25% (v/v) aqueous methanol solution with 0.178 M NH_3_(aq).

### Potential Mechanism of Negative Ion Formation

Negative ions could potentially form close to the emitter tip, throughout the plume, in the desolvation line, and in the vacuum region of the mass spectrometer. The majority of negative droplets—observed in positive electrospray—were produced near the emitter and tended to be relatively large.^11,12^ It is also possible that negatively charged nanodroplets are formed in the later stages of electrospray, giving rise to negative ions. The development of axial charge gradients in electrospray droplets via electrophoretic charge separation was suggested to occur in electrospray droplets,^40^ and such separation may precede the formation of negative droplets. The symmetric jets observed in FIDI^15^ and closer to a collector^16^ may produce nanodroplets with opposite polarities, preceding the emergence of both positive and negative ions.

In the mid-20th century, Dodd developed a theory of statistical spray droplet charging.^41^ This theory was later used to explain ion formation in the absence of electricity, as in the case of thermospray ionization,^42^ sonic spray ionization,^35,36^ bubbles and droplets ejected from the liquid surface,^43^ surface acoustic wave ionization,^44^ and zero-volt paper spray ionization.^45^ A similar mechanism may also contribute to the formation of negative ions from droplets produced by positive electrospray.

Recently, Xia et al.^46^ visualized the charge separation process in droplets, leading to the formation of positively and negatively charged droplets, analogous to the Lenard effect,^47,48^ where charge separation occurs during liquid fragmentation under atmospheric conditions. It is also possible that the “bag mechanism”—described earlier by Zilch et al.^49^—can cause the droplets to deform and break apart, releasing smaller charged species. This can promote charge separation, leading to the formation of both positive and negative droplets. These mechanisms can describe ion formation in the presence of nebulizing gas. However, a different mechanism may be needed to explain the formation of negative ions in the absence of the nebulizing gas.

The combined effect of ion dispersion due to space charge and aerodynamics, frequent collisions of ions with gas molecules (short mean free path),^50,51^ and diffusion leads to the loss of most ions produced within the electrospray plume in the atmospheric pressure region. Additional ion losses inside the transfer capillary have been attributed to space charge, diffusion, and turbulence.^52^ Ion production can be promoted by conductive heating of the aerosol passing through the transfer capillary, which accelerates the solvent evaporation/droplet fission cycle. Besides fission of highly charged droplets in the atmospheric region, alternative mechanisms may also lead to eventual negative ion formation as the charged aerosol travels toward and into the MS inlet. For instance, droplets can deposit sample liquid on the inner wall of the desolvation line. Ions could be produced either directly (cf. ref 53) or via emission of micro and nanodroplets that continue their journey toward the vacuum compartment. This mechanism could explain the formation of ions of both polarities from both charged and neutral droplets.

Atmospheric pressure ion sources may introduce relatively large droplets to the desolvation line, where ions can be formed, for example, via desorption from the liquid layer on metal surfaces. This process can contribute to MS signals, partly compensating for the low efficiency of plume droplet desolvation, ion production, or transmission. Assuming that a two-step ionization process—droplet production by electrospray, followed by charging en route to a mass analyzer—is plausible, it could also explain ion formation in other techniques involving the introduction of liquids to the mass spectrometer’s inlet. Some of the ions observed in desorption electrospray ionization MS (cf. ref 54) may possibly form through a similar mechanism as they travel to the MS through the transfer line. In inlet ionization, ions are formed after a liquid sample is directly deposited inside the desolvation line.^55^ Similarly, neutral microdroplets can be lightly charged by an induction electrode prior to their introduction into the mass spectrometer,^56^ suggesting ionization in the desolvation line. Ions of different polarities may form in the desolvation line irrespective of the voltage applied to the electrospray emitter.

Charging by the electric fields present in the high vacuum region of the mass spectrometer may facilitate ionization from sufficiently small droplets. A fraction of neutral or weakly charged liquid droplets can enter deeper sections of mass spectrometers (cf. refs 57, 58 ). Thermodynamic (e.g., droplet freezing) and kinetic (e.g., low evaporation and sublimation rates relative to the time scale of their travel to the analyzer) considerations hinder timely ion formation from these droplets.

## Conclusions

We show that ESI operated with positive voltage produces negative species, including desolvated negative ions. Direct MS detection of desolvated negative ions from standard positive electrosprays has not been reported, despite evidence of negatively charged droplets. Our results provide additional insight into the mechanism driving the pulsating electrospray. Most of the positive species seem to stay close to the spray axis. This is the region of the spray that also includes the trajectories of larger droplets with limited mobility. On average, the spray appears to be neutral at the outskirts, but positive and negative species alternately hit the counter electrode. These current oscillations on the external rings suggest that the standard explanation of spray current oscillation must be reconsidered. While overlapping charged aerosol clouds account for most of the signal, negative species also contribute to the current minima. Although the observation of negative spray current is counterintuitive, it perfectly aligns with droplets moving backward toward the electrospray meniscus.^11,12^ It is particularly striking that negative ions—traveling against the electric field—can be detected by MS when positive voltage is applied to the electrospray emitter. Several mechanisms potentially leading to the formation of negative ions are plausible, including asymmetric fission of negative droplets, symmetric fission of neutral droplets, and inlet ionization.

## Supplementary Material



## Data Availability

Data for this article are available from the corresponding author upon reasonable request.

## Notes

Additional figures S1–S8: observation of a returning droplet in paper spray; the full set of current measurements on the rings; laser-induced image of a typical electrospray plume; typical spray current measurement on ring #3; positive-ion mass spectra and currents; comparison between the signal intensity for negative ions measured by the MS in negative SIM mode with the electrospray emitter biased at +3.8 kV; with no voltage applied to the electrospray (PDF)
