# Supplementary material for: Negative Gaseous Ions in Positive-Voltage Electrospray Ionization Mass Spectrometry
Source: Anal Chem. 2026 Mar 3;98(10):7772–9. doi: 10.1021/acs.analchem.5c08108 (PMC13000871; doi:10.1021/acs.analchem.5c08108)
Supplement: Supplementary file 1 [file ac5c08108_si_001.pdf]

SUPPORTING INFORMATION

Negative Gaseous Ions in Positive-Voltage Electrospray Ionization Mass Spectrometry

Xing-Bo Wang[1#], Ochir Ochirov[1#], Bo-Cheng Ke[1], Noor Hidayat Abu Bakar[1], Chamarthi Maheswar Raju[1], Ioan Marginean[2*], Pawel L. Urban[1*]

[1] *Department of Chemistry, National Tsing Hua University*

101, Section 2, Kuang-Fu Rd., Hsinchu, 300044, Taiwan

[2] *School of Criminal Justice, University of Baltimore*

10 W Preston St, LAP 515, Baltimore, MD 21201, USA

[#] These authors contributed equally to this work.

* Corresponding authors:

I. Marginean (imarginean@ubalt.edu)

P.L. Urban (urban@mx.nthu.edu.tw)

Table of Contents

Figures S1-S8……………………………………………………………….S-2 – S-9

ADDITIONAL FIGURES

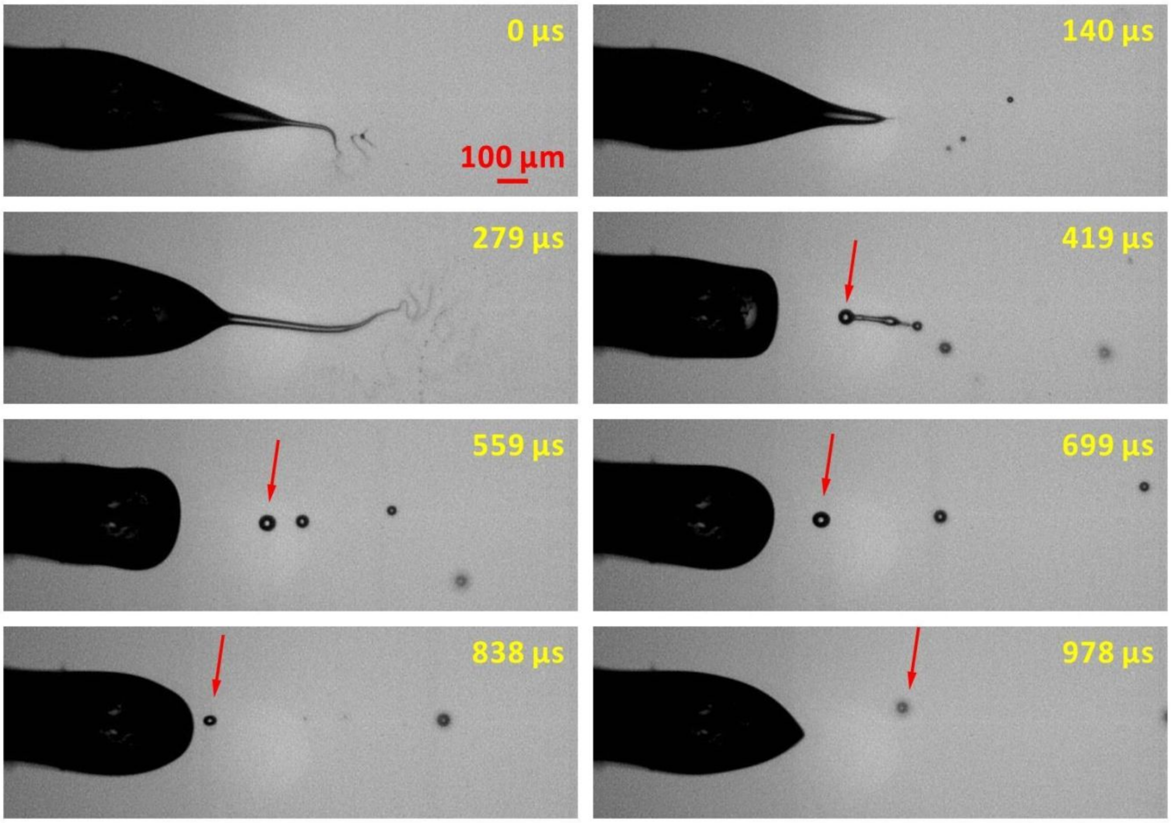

Figure S1. Observation of a returning droplet in paper spray ionization. The red arrow points to a droplet separating from the spindle, returning to the meniscus, touching the meniscus, and then moving forward. The sprayer had a triangle shape (15 × 10 mm, height × base), and was made of filter paper. Sprayed solution: 60 µL of 75% methanol (v/v) in water. Voltage: +4.5 kV. Distance from the sprayer tip to counter electrode: 10 mm. Frame rate: 78668 fps. Shutter speed: 289 ns.

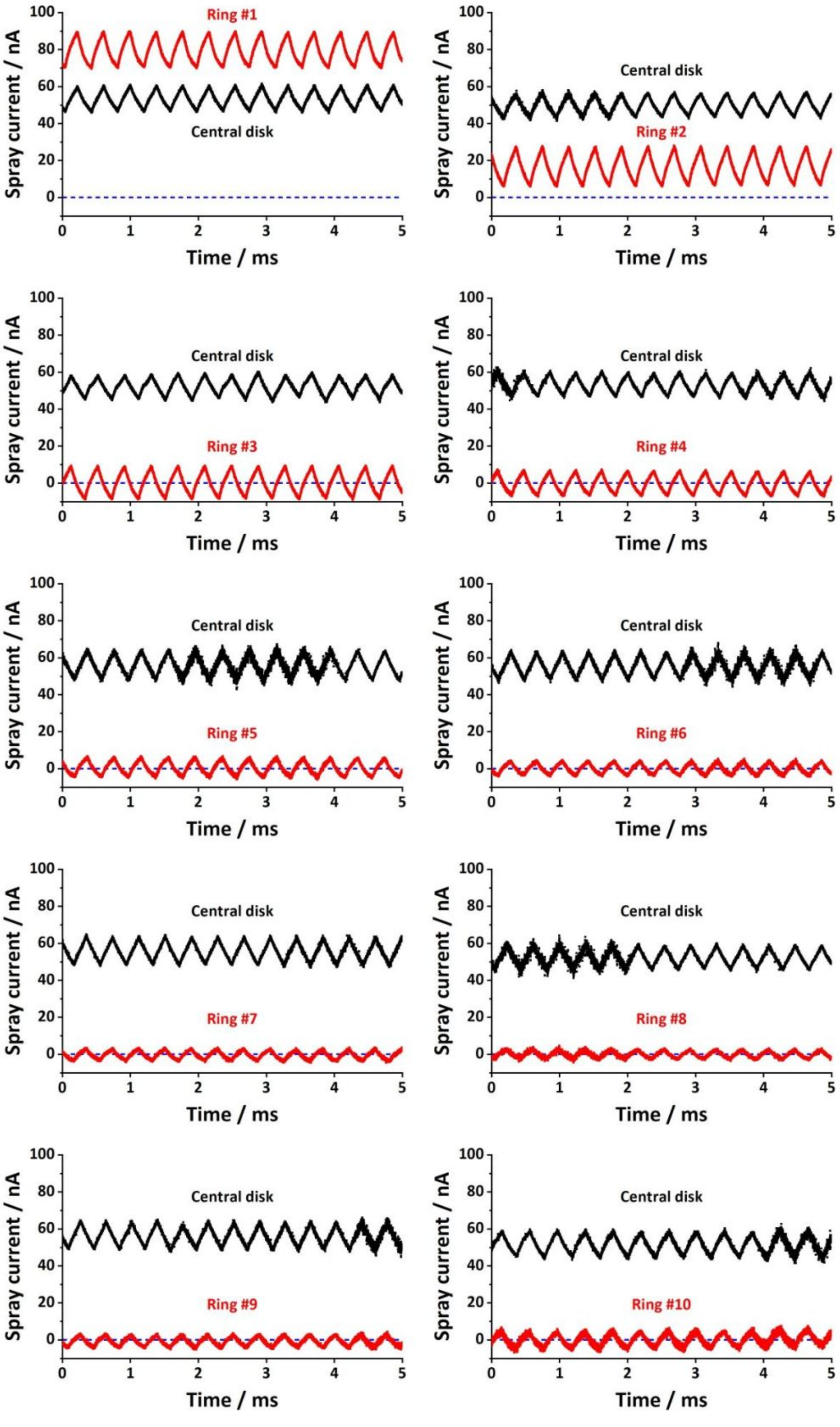

Figure S2. The full set of paired spray current measurements: the central disk paired with the respective ring from #1 to #10. ESI capillary constant voltage: +3 kV. Sample: aqueous methanol (25%, v/v) with 0.5% (v/v) formic acid. Sample flow rate: 8 μL min^{-1}. The distance between the ESI capillary tip and the structured Faraday plate: 10 mm.

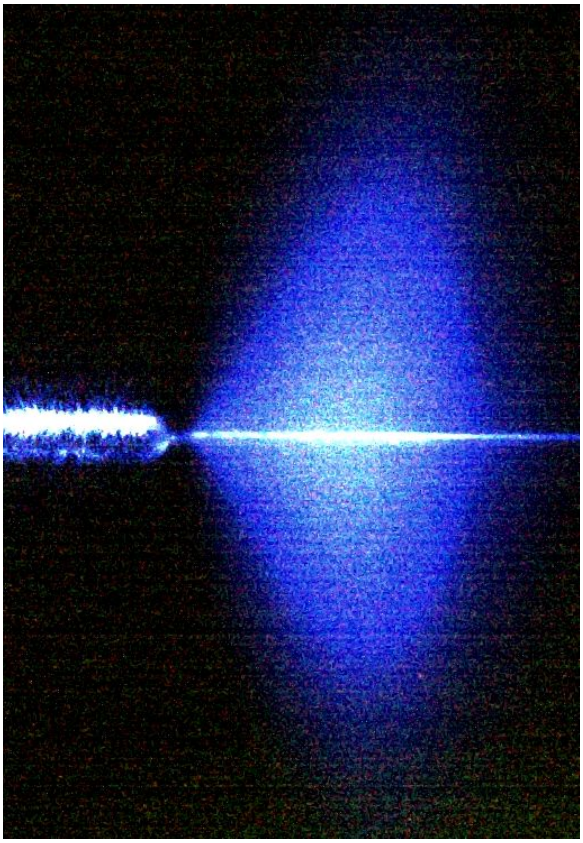

Figure S3. Image of a typical electrospray plume captured with laser illumination during current measurements on the structured Faraday collector: aqueous solution with 25% (v/v) methanol and 0.5% (v/v) formic acid; flow rate 8 μL min^{-1}; capillary voltage +3 kV; distance between the capillary tip and the electrode 10 mm. Note that the plume has a sufficiently wide angle to allow electrosprayed charged species to reach the outermost concentric rings. Moreover, the intensity of the scattered light reflects the spatial distribution of charged droplets, exhibiting a maximum at the center of the plume and gradually decreasing toward the periphery.

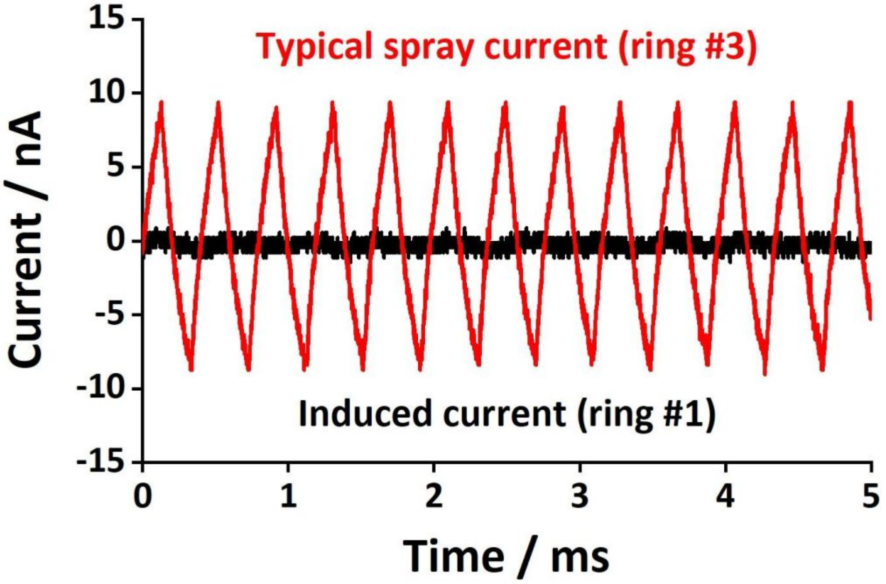

Figure S4. Typical spray current measurement on the ring #3 (red trace) compared to induced current on the ring #1 (black trace) when a sinusoidal signal was applied to the central disk. The sinusoidal wave was meant to simulate the current typically measured on the central disk (frequency of 2.0 kHz, a DC bias of 65 nA, and a peak-to-peak amplitude of 20 nA). The induced current in ring #1 had a relatively small peak-to-peak amplitude of 2.4 nA, while the spray current in ring #3 had a peak-to-peak amplitude of 18.8 nA. This indicates that the majority of the current measured on ring #3 can be attributed to the electrospray. The currents induced in electrodes farther apart from the central disk had similar peak-to-peak amplitudes: 2.2 nA for ring #5, 2.4 nA for ring #10.

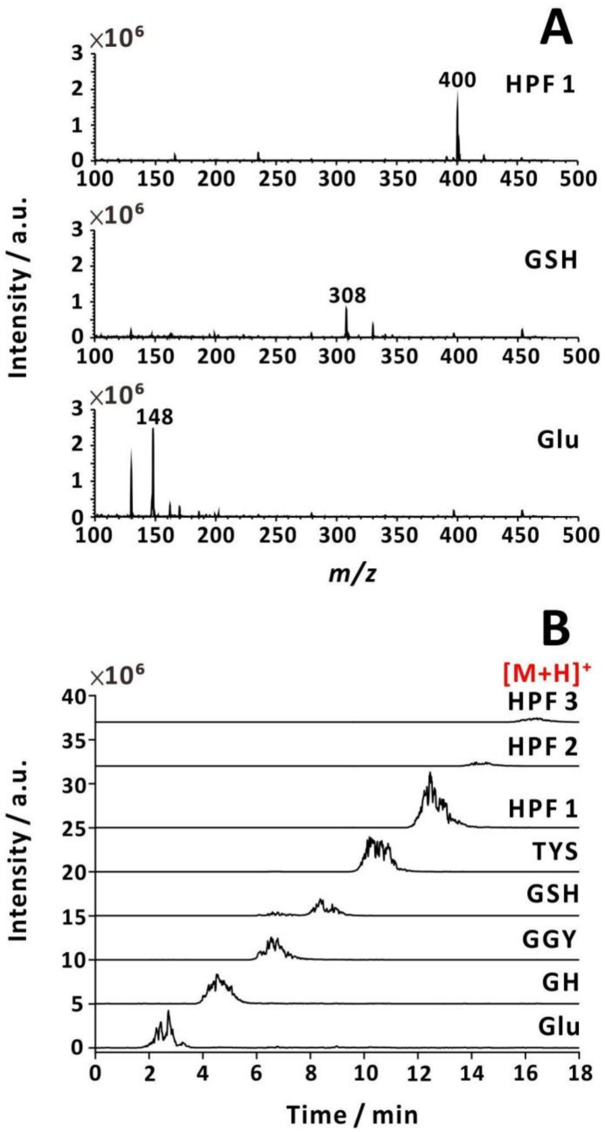

Figure S5. Positive-ion mass spectra (A) and currents recorded in SIM mode (B). Positive voltage (+3.8 kV) was applied to the emitter, and no nebulizing gas was used. Analytes: (1) HPF (*m/z* = 400); (2) GSH (*m/z* = 308); (3) L-glutamic acid (*m/z* = 148). Sample flow rate: 35 μL min^{-1}. Sample solution: 80 μM analyte in 25% (v/v) aqueous methanol solution with 0.178 M NH_3(aq).

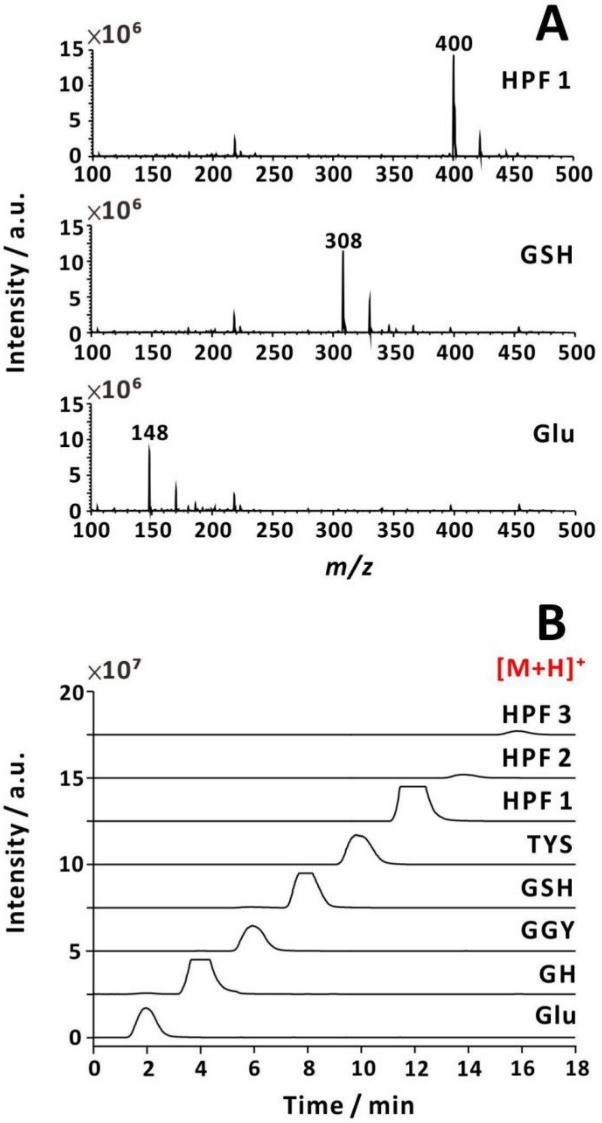

Figure S6. Positive-ion mass spectra (A) and currents recorded in SIM mode (B). Positive voltage (+3.8 kV) was applied to the emitter, and nebulizing gas was used (40 psi). Analytes: (1) HPF (*m/z* = 400); (2) GSH (*m/z* = 308); (3) L-glutamic acid (*m/z* = 148). Sample flow rate: 50 µL min^{-1}. Sample solution: 80 µM analyte in 25% (v/v) aqueous methanol solution with 0.178 M NH_3(aq).

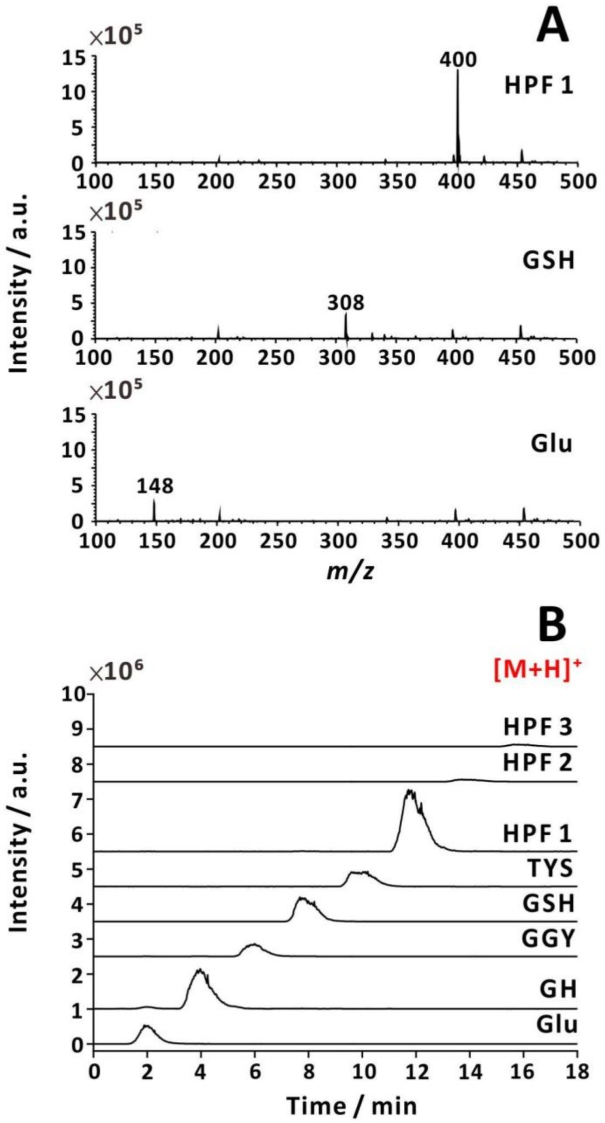

Figure S7. Positive-ion mass spectra (A) and currents recorded in SIM mode (B). No voltage (0 kV) was applied to the emitter, and nebulizing gas was used (40 psi). Analytes: (1) HPF (*m/z* = 400); (2) GSH (*m/z* = 308); (3) L-glutamic acid (*m/z* = 148). Sample flow rate: 50 µL min^{-1}. Sample solution: 80 µM analyte in 25% (v/v) aqueous methanol solution with 0.178 M NH_3(aq).

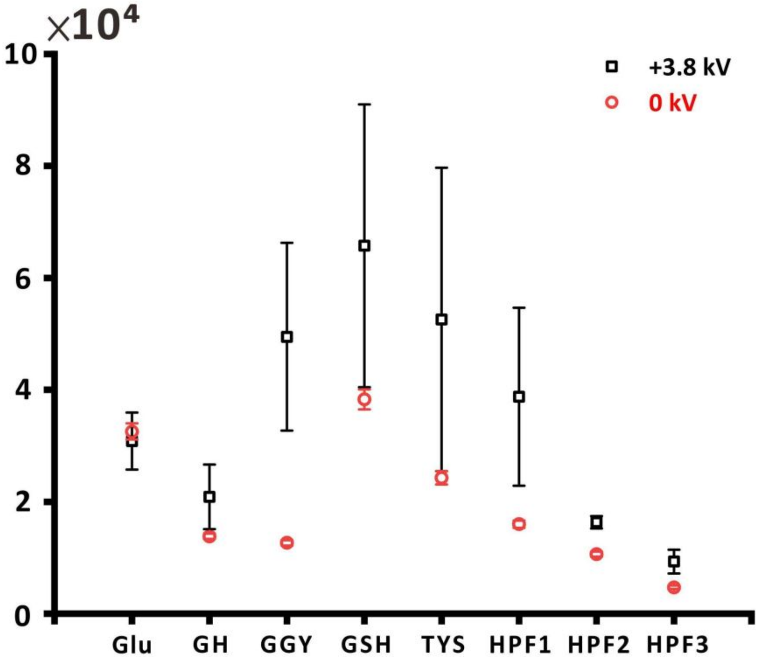

Figure S8. Comparison between the signal intensity for negative ions measured by the MS in negative SIM mode with the electrospray emitter biased at +3.8 kV (black) and with no voltage applied to electrospray (red). Nebulizing gas pressure: 40 psi. Error bars correspond to standard deviations calculated based on three experiments. Analytes: L-glutamic acid (*m/z* = 146), GH (*m/z* = 211), GGY (*m/z* = 294), GSH (*m/z* = 306), TYS (*m/z* = 368), HPF 1 (*m/z* = 398), HPF 2 (*m/z* = 779), HPF 3 (*m/z* = 1161).
